# Bispecific T-Cell Engaging Antibodies Against MUC16 Demonstrate Efficacy Against Ovarian Cancer in Monotherapy and in Combination With PD-1 and VEGF Inhibition

**DOI:** 10.3389/fimmu.2021.663379

**Published:** 2021-04-14

**Authors:** Oladapo O. Yeku, Thapi Dharma Rao, Ian Laster, Artem Kononenko, Terence J. Purdon, Pei Wang, Ziyou Cui, Hong Liu, Renier J. Brentjens, David Spriggs

**Affiliations:** ^1^ Division of Hematology-Oncology, Massachusetts General Hospital, Boston, MA, United States; ^2^ Department of Medicine, Massachusetts General Hospital, Boston, MA, United States; ^3^ Department of Medicine, Memorial Sloan Kettering Cancer Center, New York, NY, United States; ^4^ Eureka Therapeutics Inc., Emeryville, California, United States

**Keywords:** ovarian cancer, bispecific antibodies, bispecific engagers, MUC16, MUC16ecto, VEGF, angiogenesis, immune checkpoint blockade

## Abstract

Immunotherapy for ovarian cancer is an area of intense investigation since the majority of women with relapsed disease develop resistance to conventional cytotoxic therapy. The paucity of safe and validated target antigens has limited the development of clinically relevant antibody-based immunotherapeutics for this disease. Although MUC16 expression is almost universal in High Grade Serous Ovarian Cancers, engagement of the shed circulating MUC16 antigen (CA-125) presents a theoretical risk of systemic activation and toxicity. We designed and evaluated a series of bispecific tandem single-chain variable fragments specific to the retained portion of human MUC16 ectodomain (MUC16^ecto^) and human CD3. These MUC16^ecto^- BiTEDs retain binding in the presence of soluble MUC16 (CA-125) and show cytotoxicity against a panel of ovarian cancer cells *in vitro*. MUC16^ecto^- BiTEDs delay tumor progression *in vivo* and significantly prolong survival in a xenograft model of ovarian peritoneal carcinomatosis. This effect was significantly enhanced by antiangiogenic (anti-VEGF) therapy and immune checkpoint inhibition (anti-PD1). However, the combination of BiTEDs with anti-VEGF was superior to combination with anti-PD1, based on findings of decreased peritoneal tumor burden and ascites with the former. This study shows the feasibility and efficacy of MUC16^ecto^- specific BiTEDs and provides a basis for the combination with anti-VEGF therapy for ovarian cancer.

## Introduction

Epithelial Ovarian Cancer (EOC) continues to be a highly lethal disease (1). This is due in large part to the evolution of a multi-drug resistant phenotype in virtually all women with recurrent disease (2). Immunotherapeutic modalities represent an opportunity to achieve durable clinical responses in this subset of patients. This could be accomplished by harnessing endogenous tumor-reactive cytotoxic T-cells. In one study, the presence of tumor-infiltrating T-cells (TILs) corresponded with a 5-year overall survival of 38% compared to 4% in patients without TILs (3). Currently, there are ongoing efforts to harness the immune system against ovarian cancer using vaccine therapy (4), immune checkpoint blockade (5), adoptive cellular therapy (6), and antibody-drug conjugates (7).

Bispecific T-cell engagers (BiTEs) are antibodies consisting of two tandem-linked variable heavy (V_H_) and light (V_L_) chains with specificity for a tumor-associated antigen (TAA) and frequently the cluster of differentiation 3 (CD3) complex expressed on T-cells (8). Bispecific tandem linked single chain variable fragments (taFv, scFv_2_) are a subtype of BiTEs that involve two covalently linked single chain variable fragments (scFv) (9). One scFv is specific for a TAA, and the other binds to the CD3 subunit of the T-cell receptor (10). Upon administration, taFv, scFv_2_ BiTEs redirect polyclonal T cells to the tumor, whereupon the formation of immunologic synapses leads to tumor-specific cytolysis (11). These redirected T-cells function independently of T-cell innate antigen-specific receptor (TCR) recognition and are capable of serial cytotoxicity (11) and secretion of inflammatory cytokines (12). Unlike other T-cell based approaches like Chimeric Antigen Receptor (CAR) T-cell therapy, taFv, scFv_2_ BiTEs do not require T-cell genetic engineering (13) and are not restricted by the continued expression of the TAA, limiting the potential for immune escape *via* downregulation of the target antigen. BiTEs have been shown to be effective for the management of CD19+ hematologic tumors (14) and BiTEs against antigens expressed on solid tumors such as WT1 (15), ROR1 (16), PSMA (17, 18), and B7H6 (19) have been described, however, none of these have been approved for clinical use.

The MUC16 protein is a heavily glycosylated member of the mucin family with normal Mullerian tissue expression and is overexpressed on High Grade Serous Epithelial Ovarian Cancer cells (HGSOC) (20). MUC16 is post-translationally cleaved into a soluble antigenic fragment from the tandem repeat region (detected as CA-125) and a retained extracellular fragment- termed MUC16^ecto^ with independent pro-oncogenic properties (21). The majority of antibody based anti-MUC16 clinical therapeutics target the shed portion of MUC16 (22) which may limit their specificity as targeted immunotherapy. We have previously reported a murine monoclonal antibody specific to MUC16^ecto^ (23) and validated scFv derived from this antibody using CAR-based immunotherapy (6, 24, 25).

In this report, we describe the generation and validation of a bispecific tandem linked single chain variable fragment directed to MUC16^ecto^ (henceforth termed MUC16^ecto^-BiTEDs) derived from a human phage display library. These human scFv are advantageous for clinical therapeutics due to a decreased risk of human anti-mouse antibody (HAMA) reactions (26). MUC16^ecto^-BiTEDs are specific for MUC16^ecto^ *in vitro* and are shown to decrease tumor progression and prolong survival in tumor-bearing mice. We explore potential mechanisms for treatment failure and evaluate potential combination with anti-PD-1 and anti-VEGF monoclonal antibodies. Validation of our MUC16^ecto^- BiTEDs expands the scope of MUC16-directed immunotherapy and sets the foundation for combinatorial strategies in ovarian cancer.

## Materials and Methods

### Human Phage Display Panning

E-ALPHA^®^ human phage display library was used to screen for clones that specifically bind to MUC16^ecto^. Independent panning was carried out using 15 different phage sub-libraries. Individual scFv phage clones positive for MUC16^ecto^ were determined by FACS and the clones that possessed unique DNA coding sequences were subjected to further characterization. Positive phage clones were further validated for binding to MUC16^ecto^ overexpressing HEK293 cells. For phage display screening, unmodified HEK293, HEK293 expressing MUC16 (HEK-MUC16WT) or HEK293 cells expressing mutant MUC16 (HEK293-MUC16mut) were used. For FACS screening, phage clones were incubated with MUC16^ecto^ overexpressing HEK293 cells, then with anti-M13 mouse antibody. APC-labeled anti-mouse IgG secondary antibody was added to the reaction after washing. Binding was measured by FACS and expressed as mean fluorescence intensity (MFI). Cells incubated with secondary antibody alone, M13 K07 helper phage, and cells only were used as negative controls.

### Expression, Production, and Purification of MUC16^ecto^-BiTEDs

**Figure 1:**
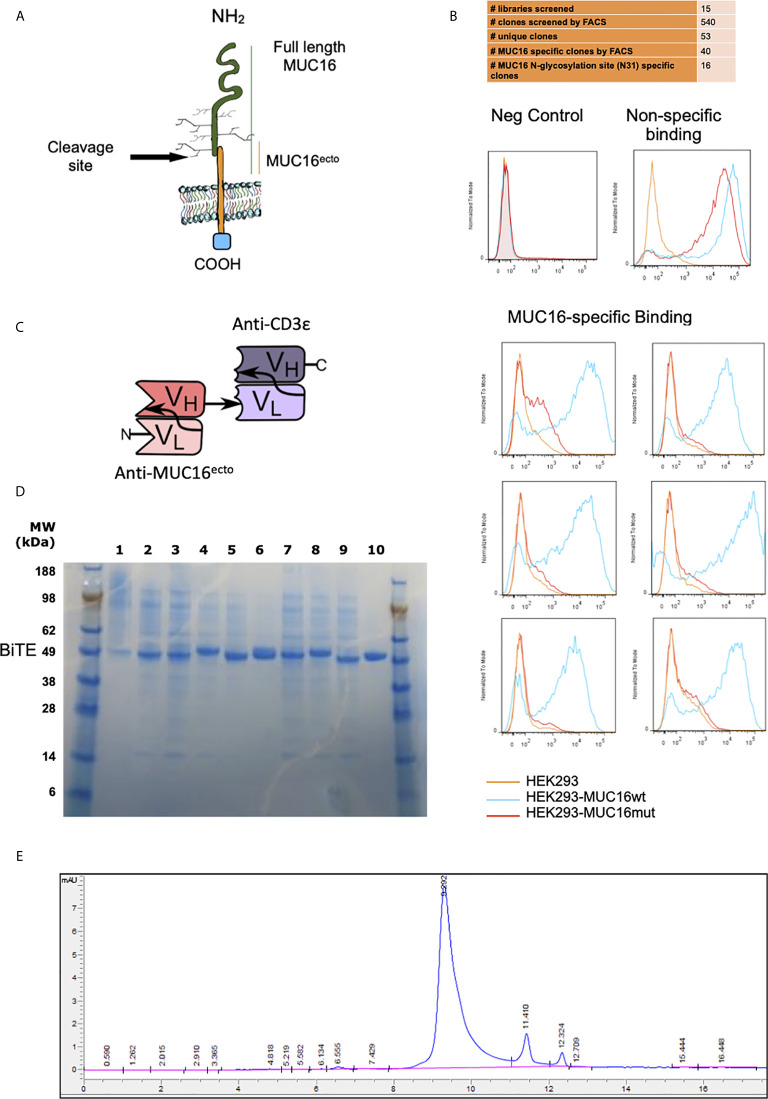
Screening and identification of human MUC16^ecto^ specific antibodies. **(A)** Schematic representation of MUC16 antigen and the c114 retained ectodomain (MUC16^ecto^) which serves as the antibody target. **(B)** Phage display binding analysis by FACS. Only targets that bind to MUC16^ecto^ (blue) and not the mutant MUC16 (orange) were selected for sequencing and further development. Examples of negative control (top left) and non-specific binding (top right) are shown. **(C)** Schematic representation of MUC16^ecto^ BiTE configuration. **(D)** Purity of CD3ϵ-congugated MUC16^ecto^ -specific BiTEDs were validated by SDS-PAGE. Expected band size 50-55 KDa. **(E)** MUC16^ecto^-BiTEDs purity and structural integrity evaluated by SE-HPLC. The SE-HPLC analysis demonstrates the correct apparent molecular weight with the majority of the protein in monomeric form.

MUC16^ecto^-BiTEDs consist of one arm anti-MUC16 scFv at the N terminus, and the other arm consists of an anti-CD3ϵ scFv at the C terminus (**Figure 1C**). The two scFv are joined covalently through a generic linker 3*G4S (GGGGSGGGGSGGGGS). The anti-CD3ϵ specific single chain antibody is derived from the mouse monoclonal antibody L2K. The deimmunized L2K has been previously reported (27). DNA fragments coding for the MUC16 scFv and the anti-human CD3ϵ scFv were synthesized and subcloned into EUREKA Therapeutics mammalian expression vector (pGSN- Hyg) using routine cloning methods. To facilitate purification and detection, a hexahistidine (His) tag was inserted downstream of the bispecific engager at the C-terminal end (28). The sequence-validated construct was transduced into Chinese hamster ovary (CHO) cells in serum free media. CHO cell supernatants containing secreted bispecific engager molecules were collected and purified using a HisTrap HP column (AKTA FPLC, GE healthcare). Briefly, conditioned CHO cell supernatants were clarified and loaded onto the column containing low concentrations of imidazole (20 mM) and then subjected to an isocratic high imidazole concentration elution buffer (500 mM) to elute the bound the bispecific antibody protein. The eluted MUC16^ecto^-BiTEDs was reconstituted in PBS for stock for single use aliquots prior to freezing. The final MUC16^ecto^-BiTEDs products were subjected to SDS-PAGE and size exclusion chromatography-HPLC for QC.

### Cell Lines and Cytotoxicity

For cytotoxicity assays, SKOV3 (MUC16^Neg^), SKOV3-MUC16^ecto^ (MUC16^Pos^), OVCAR3 (MUC16^Pos^), OVCAR432 (MUC16^Pos^), and SKOV8 (MUC16^Pos^) cell lines were used. For luciferase-based cytotoxicity assays, imaging, and survival assays; SKOV3 modified to express MUC16^ecto^ and the luciferase gene (SKOV3-MUC16^ecto^-Luc), and wild type isogenic SKOV3-Luc cells were used. All human ovarian cancer cell lines were maintained in RPMI (Invitrogen, Grand Island, NY, USA) supplemented with 10% heat-inactivated fetal calf serum (FBS), 100 U/ml penicillin and streptomycin (P/S), and 2mM L-glutamine. Cells were validated using karyotype analysis and routinely checked for mycoplasma contamination. Flow cytometry analyses was performed using Gallios Flow Cytometer with Kaluza software (Beckman Coulter, Brea, CA, USA). MUC16^ecto^ expression was detected using APC-conjugated anti-Muc16 antibody. Human T cells were derived from fresh blood-derived leukocyte concentrate (Leukopack) obtained from the New York Blood Center, mononuclear cells were separated using density gradient centrifugation with Accu-prep (axis-Shield PoC AS). T cells were isolated, activated and expanded with PHA (Sigma Aldrich, St Louis, MO) at a concentration of 2x10^6^/ml. T cells were cultured in RPMI supplemented with 10% fetal calf serum, 100 U/ml penicillin and streptomycin, and 2mM L-glutamine, in the presence of 100 IU/ml recombinant human IL-2 (Proleukin). Viable cells were enumerated using flow cytometry and counting beads (Ebioscience). For LDH-based cytotoxicity, tumor cells and activated T-cells were cocultured 1:1 in the presence of 0.2μg/ml of the relevant BiTEDs for 16 hrs. LDH release assay was used to quantify dead cells according to the manufacturers protocol. For luciferase-based cytotoxicity assays, activated donor T-cells were cocultured with SKOV3-Luc, SKOV3-MUC16^ecto^-Luc or OVCAR3-Luc at the indicated effector: target ratios in the presence of 0.5μg/ml of BiTEDs for 48 hrs, and subsequently mixed with luciferase assay reagent (Promega). Luminescence of the lysates was analyzed using a plate spectrophotometer. Unless otherwise stated, all cytotoxicity experiments were performed with at least four separate donors and repeated a minimum of three times. Specific cytolysis was calculated using the formula; % specific lysis = 100 × (sample lysis –spontaneous lysis)/(maximal lysis –spontaneous lysis).

### SDS-PAGE Validation

Candidate anti-CD3ε bispecific diabodies were evaluated using SDS-PAGE. Samples were run using the NuPAGE^®^ Novex^®^ Bis-Tris gel (4-12%) and NuPAGE^®^ MEX x1 running buffer under reducing conditions at 70°C for 10 mins. The expected band size was 50-55 KDa.

### Immunoprecipitation

For immunoprecipitation experiments, 1.5 μM of affinity-purified BTM protein (Fc fragment of heavy chain fused with the antigen) was added to equal amounts of MUC16^ecto^-BiTEDs in PBS buffer, and the mixture was incubated with rotation in the presence of Protein G agarose. The immunocomplex was adsorbed onto 25 μl suspension of protein G-agarose beads (millipore) (pre-washed 3 times with PBS buffer) by incubating the mixture for 90 minutes at 4°C. The beads were washed 3 times with 600 μl of PBS buffer. Finally, the beads were resuspended in 30 μl of 0.1 M Glycine pH, 2.7 for elution of the complex. The eluate was mixed with SDS sample buffer, heated for 8 min, and the proteins were separated by NuPAGE™ 4-12% Bis-Tris Protein Gels, (Life Technology, NP0335BOX). The protein bands were detected in the gel by Coomassie Blue staining.

### Kinetic Analysis of BiTEDs Binding to MUC16^ecto^

Kinetic analysis of EXT170-8 BiTEDs and MUC16 BTM protein (55mer highly conserved ectodomain region of MUC16) was performed on a BiaCore X100 instrument loaded with an NTA sensor chip. Briefly, His-tagged EXT170-8 BiTEDs were immobilized onto NTA sensor chip at a concentration of 20 μg/ml, and MUC16 BTM protein (35 kDa) was injected at concentrations of 570, 285, 142.5, 71.25, and 35.625 nM (20, 10, 5, 2.5 and 1.25 ul/ml). The raw data was analyzed using the kinetic model 1:1 binding by Biacore X100 evaluation software.

### ELISA

96-well clear, flat-bottom plates (Thermo Scientific, 14-245-153) were coated with 1ug/ml BTM diluted in 0.1M sodium bicarbonate coating buffer (pH 8.0) overnight at 4C. Each well was washed with PBS-T (0.05% Tween-20) and subsequently blocked with PBS+2% BSA at room temperature for 1 hour. Wells were then washed with PBS-T before the addition of either biotinylated BiTEDs (Biotin labeling kit; Roche, 11418165001) or biotinylated MUC16^ecto^-BiTEDs with free rhCA-125 (R&D systems, 5609-MU). Ratios of 1:1, 5:1, and 10:1 rhCA-125 to coated BTM were used. Following a one-hour incubation at room temperature on a plate rocker, wells were washed with PBS-T. Streptavidin-HRP A (R&D systems, 890803) at 1:200 in PBS was then added to each well and incubated for 1 hour at room temperature under foil. Each well was washed with PBS-T before the addition of TMB ELISA Peroxidase Substrate (Rockland) and allowed to develop for 20 minutes, protected from light, at room temperature. Reactions were then quenched with 0.6N sulfuric acid. Wavelengths of 450nm and 540nm (for plate refraction correction) were measured *via* SpectraMax iD3 Microplate Reader. VEGF detection was performed on OVCAR3 cells cultured in 6-well plates cultured for 48 hours in complete RPMI according to manufacturer’s instructions (R&D systems, DVE00).

### FACS Analyses

Flow cytometric analyses were performed using Gallios Flow Cytometer with Kaluza software (Beckman Coulter, Brea, CA, USA). Muc16^ecto^ expression was detected using APC-conjugated anti-Muc16 antibody (Memorial Sloan Kettering Cancer Center mono- clonal antibody facility). Human cells were stained with mouse anti human CD3 (PE/APC. Thermofisher UCHT1/OKT3), PD-1 (APC. Thermofisher MIH4), TIM3 (APC. Thermofisher F38-2E2), LAG3 (APC. Thermofisher 3DS223H), Granzyme B (APC, Thermofisher GB11), CD4 (PE, Thermofisher RPA-T4), CD8 (PE, Thermofisher RPA-T8), VEGF (PE, R&D systems IC2931P), PD-L1 (APC. Thermofisher MIH1), and CD45 (APC. Thermofisher HI30). Tumor, splenocytes or peritoneal cells were pelleted and washed 3 times with FACS buffer (PBS + 2.5% FBS). Cells were resuspended with the appropriate antibody, diluted in FACS buffer and incubated at 4°C for 30 min in the dark. The cells were subsequently washed 3 times with cold FACS buffer and resuspended in 1X DAPI prior to FACS analysis.

### Cytokine Measurement

Serum cytokines were measured from blood collected *via* retro-orbital bleeds from indicated animals and centrifuged to separate the serum fraction. Cytokine detection was performed using the MILLIPLEX MAP Human Cytokine/Chemokine, Premixed 13 Plex kit, and the Luminex IS100 system. Dedicated cytokine assays for IL-2 (abcam, ab174444) and IFN-γ (abcam, ab174443) were performed with commercial ELISA kits according the manufacturers protocol.

### Animal Imaging and In Vivo Experiments

Female NSG mice age 6-8 weeks were purchased from Jackson Laboratory, Bar Harbor, ME, USA. 3x10^6^ SKOV3- MUC16^ecto^/-Luc, or OVCAR3 tumor cells were injected intraperitoneally (i.p.) on D0, and animals were untreated, treated with T-cells intravenously (i.v) alone or treated with a combination of T-cells and 5μg MUC16^ecto^- BiTEDs on day 7. Animals in the BiTEDs treatment group received additional 5μg MUC16^ecto^- BiTEDs treatment on days 9, 11, 14, 16, and 18 for a total of 6 treatments over 2 weeks. Tumor-bearing mice were injected intraperitoneally with D-Luciferin (Goldbio Technology) (150 mg/kg) and after 10 min were imaged under isofluorane anesthesia. Bioluminescent imaging was achieved using the Caliper IVIS imaging system and analyzed with Living Image 4.0 software (PerkinElmer). Image acquisition was achieved using a 25 cm field of view, medium binning level and 60s exposure time. Animals treated with αPD-1 blocking antibody (BioLegend EH12.2H7) received 250μg injected i.p on days 7, 14, 21 and 28 (weekly injections x 4 weeks) after tumor inoculation (day 0). Animals treated with αVEGF blocking antibody (Invivo Gen, hvegf-mab1) received 5 mg/kg i.p injections on days 7, 11, 14, 18, and 21 after tumor inoculation. All mice were monitored for survival and were euthanized when showing signs of distress.

### Statistical Analysis

Survival curves were analyzed using Mantel–Cox (log-rank) test and other analysis were performed using unpaired two-tailed T test (p value <0.05 considered as significant). All calculations were performed using Prism 7 (GraphPad) software.

## Results

### Human Phage Panning and Identification of MUC16^ecto^ Specific Clones

To mitigate the development of murine-anti-human antibodies, the E-ALPHA^®^ human phage display library was used to screen for clones that specifically bind to the retained portion of MUC16 (**Figure 1A**). Positive clones were screened by flow cytometry using HEK293 cells modified to overexpress MUC16^ecto^ (MUC16^c114^). Clones bound to MUC16^ecto^ but not the mutant form were selected for further development into bispecific engagers (**Figure 1B**). A total of 15 libraries were screened, which yielded 540 clones that were subjected to verification by flow cytometry. Of these, 53 unique clones were identified, and ultimately 16 clones showed preferential binding to MUC16^ecto^.

### Validation of MUC16^ecto^ Specific Bispecific T-Cell Engagers

**Figure 2:**
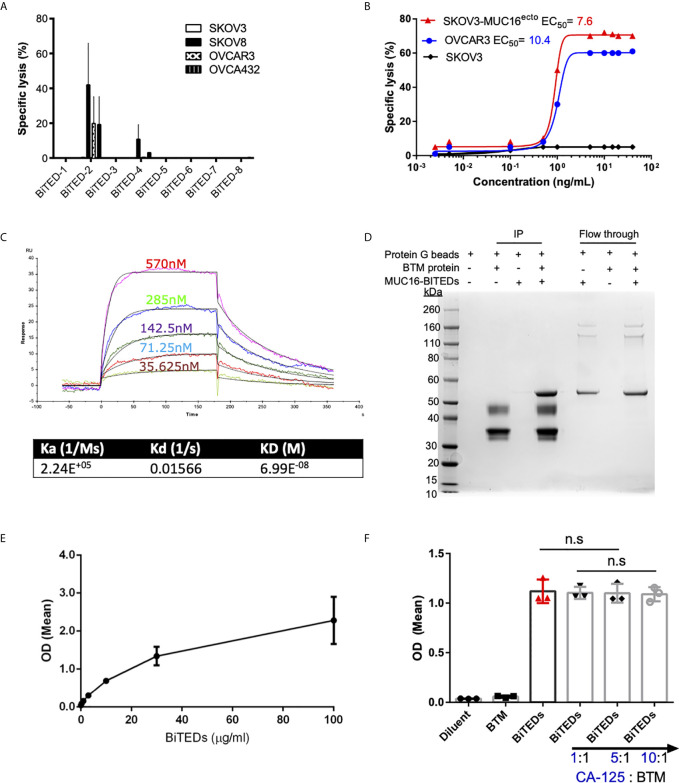
Validation of lead MUC16^ecto^-BITEDs candidate, evaluation of binding to MUC16 ectodomain and potential interference by soluble CA-125. **(A)** Evaluation of cytotoxicity *via* 1:1 E:T coculture of SKOV3, SKOV8, OVCAR3, OVCAR432 and activated T-cells with the indicated BiTEDs. **(B)** Increasing concentrations of MUC16^ecto^-BiTEDs cocultured with MUC16 positive SKOV3 (SKOV3-MUC16^ecto^), OVCAR3, and MUC16 negative SKOV3 cell lines, and PBMC for 48 hrs PBMC (E:T = 10:1). Cytotoxicity determined by LDH release assay. **(C)** Kinetic analysis was performed to determine the dissociation constant of MUC16^ecto^-BiTEDs to the highly conserved 55mer MUC16 ectodomain. **(D)** BTM protein was immunoprecipitated (IP) with MUC16^ecto^-BiTEDs (lane 4). Control conditions; Protein G agarose beads alone (lane 1), protein G beads with BTM protein (lane 2) or with MUC16^ecto^-BiTEDs (lane 3) did not show the expected 55 KDa band representing MUC16^ecto^-BiTEDs. Unbound MUC16^ecto^-BiTEDs but not BTM protein were detected in flow through (lanes 5-7). **(E)** ELISA showing binding of increasing concentrations of MUC16^ecto^-BiTEDs in the presence of BTM. **(F)** ELISA showing binding of diluent and MUC16^ecto^-BiTEDs in the presence of increasing concentrations of recombinant human CA-125. For **(A, B)**, data shown are pooled results from 3 independent experiments with 3 independent donors. Data are plotted as mean ± SEM. Results from **(E, F)** are pooled from 3 independent experiments. Data are plotted as mean ± SEM. ns, not significant.

Single chain variable fragments (scFv) derived from candidate phage libraries were cloned into a bispecific antibody construct with one arm expressing anti-human CD3ε scFv antibody (**Figure 1C**) in a tandem-scFv configuration. MUC16^ecto^- BiTEDs were purified and confirmed by SDS-page (**Figure 1D**). MUC16ecto-BiTEDs were expressed in CHO cells and purified using HisTrap column chromatography with purity and structural integrity demonstrated by SE-HPLC (**Figure 1E**). Most of the purified protein is a ~55 kDa monomer with minimal dimer or trimer forms. In order to narrow our BiTE candidates, we evaluated cytotoxicity against a variety of ovarian cancer cell lines (**Figure 2A**). Using a panel including SKOV3 (MUC16^neg^), SKOV8 (MUC16^pos^), OVCAR3 (MUC16^pos^), and OVCAR432 (MUC16^pos^), we identified a suitable BiTE candidate with cytotoxicity against MUC16^pos^ but not MUC16^neg^ ovarian cancer cell lines (**Figure 2A**). Of the two lead candidates, we selected BiTED-2 for further development. MUC16^ecto^-BiTEDs mediated selective cytotoxicity against MUC16 positive SKOV3 (SKOV3-MUC16^ecto^; EC_50_ = 7.6 ng/mL) and OVCAR3 (EC_50_ = 10.4 ng/mL) cells, but not the MUC16 negative SKOV3 cell lines (**Figure 2B**). To determine the binding affinity of our lead BiTED-2 to MUC16^ecto^, we used a previously validated BTM protein, a synthetic fusion protein consisting of the highly conserved extracellular portion of MUC16^c57-114^ fused to a human Fc backbone pFUSE (29, 30), to perform kinetic analysis using surface plasmon resonance. The dissociation constant (K_D_) for our lead MUC16^ecto^- BiTEDs was 69.9 nM (**Figure 2C**).

### MUC16^ecto^-BITEDs Bind to the Extracellular Domain of MUC-16 and Not Shed CA-125

In prior studies, we have shown that antibodies directed against MUC16^ecto^ are specific for the retained domain and not the cleaved portion of MUC16 (CA-125) (29). In this report, our lead-candidate selection strategy was specifically designed to identify clones that recognized the N-glycosylation site (N31) of MUC16^ecto^ (MUC16-C114) and not a mutant region (MUC16-N123) (**Figures 1A, B**). We chose to evaluate direct protein-protein binding of MUC16^ecto^- BiTEDs with MUC16^ecto^ *via* co-immunoprecipitation (**Figure 2D**). As shown in **Figure 2D**, a mixture of MUC16^ecto^- BiTEDs with BTM produced the expected protein band (lane 4, ~ 55 kDa), but not BTM without MUC16^ecto^- BiTEDs (lane 2) or vice versa (lane 3). To further validate that our BiTEDs bound to MUC16^ecto^, we performed an ELISA using plate-bound BTM. As shown in **Figure 2E**, MUC16^ecto^- BiTEDs bound to BTM in a concentration-dependent manner. Next, to evaluate if this binding could be disrupted by shed CA-125, we repeated the ELISA in the presence of increasing concentrations of CA-125. At 1:1, 5:1, and up to a 10-fold increase in CA-125 to BTM concentrations, we did not see any decrease in MUC16^ecto^- BiTEDs binding (**Figure 2F**).

### MUC16^ecto^- BiTEDs Display Specific Cytotoxicity Against a Panel of Ovarian Cancer Cell Lines *In Vitro*

**Figure 3:**
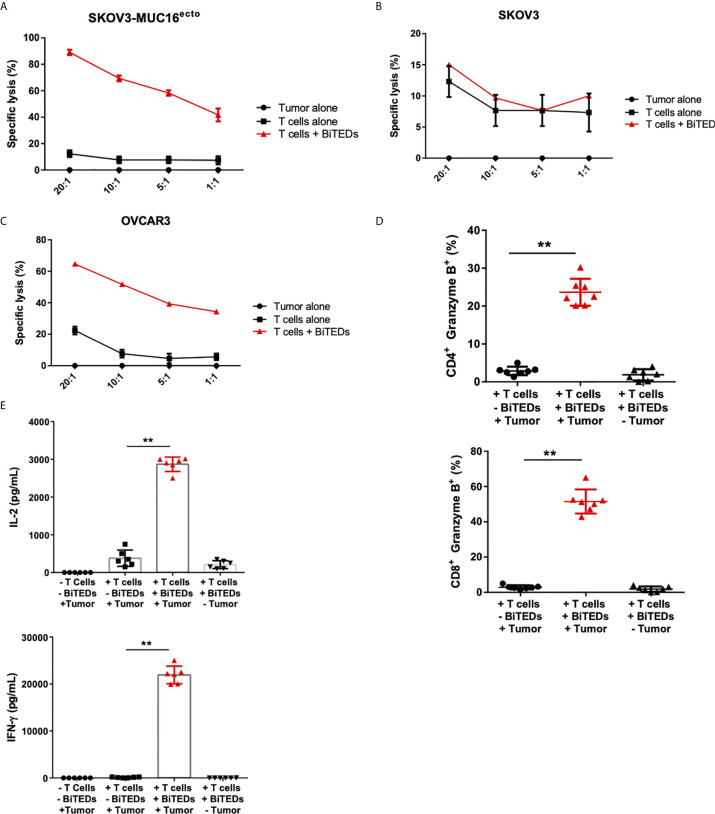
MUC16^ecto^-BITEDs display cytotoxicity against ovarian cancer cell lines *in vitro.* **(A)** Coculture of MUC16^ecto^-BITEDs and activated T-cells and SKOV3-MUC16^ecto^; **(B)** with SKOV3, and **(C)** OVCAR3 tumor cells at the indicated E:T ratios. Data are plotted as mean ± SEM. Data shown are pooled results from 3 independent experiments. At least 4 separate donors were used for each experiment. Statistics performed using unpaired two-sided T test. **(D)** Percentages of Granzyme B positive CD4 and CD8 T-cells cocultured with SKOV3-MUC16^ecto^ cells with or without MUC16^ecto^-BITEDs. **(E)** In vitro cytokine analysis of supernatants obtained from SKOV3-MUC16^ecto^ cells alone, and coculture of SKOV3-MUC16^ecto^ cells with T-cells with or without MUC16^ecto^-BITEDs. For **(D, E)**, data shown are pooled results from 5 independent experiments with at least 4 independent donors. Data are plotted as mean ± SEM. **p < 0.05.

To minimize the potential that some of the cytotoxicity observed was due to cell line-specific differences, we used isogenic SKOV3 cell lines modified to express MUC16^ecto^ (25, 31) and found dose-dependent cytotoxicity at different effector to target ratios (**Figure 3A**). Treatment of wild type SKOV3 (MUC16^neg^) cells with MUC16^ecto^- BiTEDs did not result in any significant cytotoxicity compared to incubation with T-cells alone at different E:T ratios (**Figure 3B**). OVCAR3 cells endogenously express MUC16 and shed high levels of CA-125 (32), making this cell line an ideal candidate to test the hypothesis that MUC16^ecto^- BiTEDs could mediate cytotoxicity in the presence of shed CA-125. OVCAR3 cells were effectively lysed at various E:T ratios in the presence of MUC16^ecto^- BiTEDs (**Figure 3C**). Taken together, MUC16^ecto^- BiTEDs exhibit specific cytotoxicity against MUC16^pos^ ovarian cancer cell lines, and this cytotoxicity is preserved in the presence of CA-125. To investigate the mechanisms of BiTEDs-mediated cytotoxicity, we evaluated intracellular granzyme-B and cytokine levels in T-cells cultured for 24 hours with MUC16^pos^ ovarian cancer cells with or without BiTEDs. Both CD4^+^ and CD8^+^ T-cells showed increased levels of intracellular granzyme-B (**Figure 3D**). Similarly, intracellular T-cell IL-2 and IFN-γ were significantly elevated in coculture conditions containing MUC16^ecto^- BiTEDs (**Figure 3E**). MUC16^ecto^- BiTEDs cocultured with T-cells in the absence of tumor cells did not display increased granzyme B, IL-2 or IFN-γ (**Figures 3D, E**).

### MUC16^ecto^-BiTEDs Delay Progression of Metastatic Ovarian Cancer *In Vivo*

**Figure 4:**
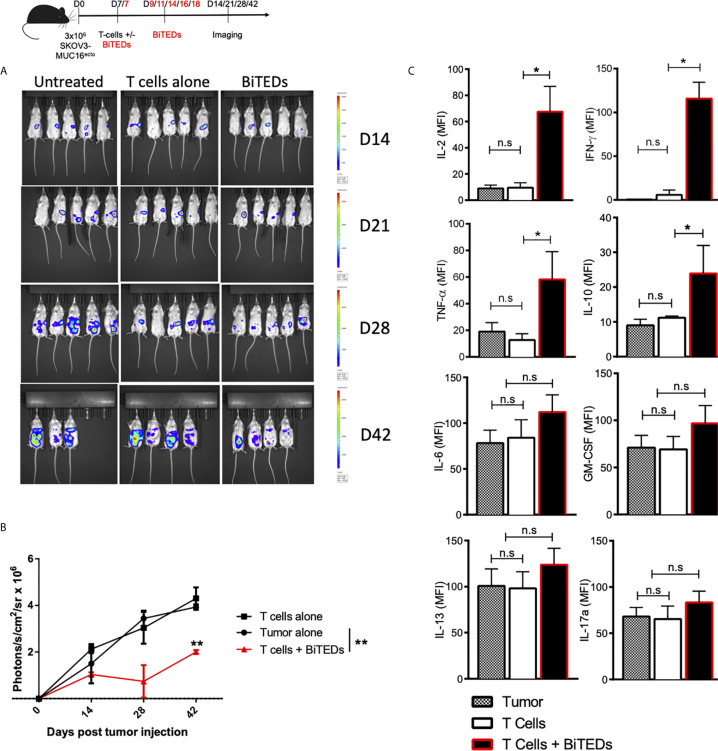
MUC16^ecto^ specific BiTEDs delay ovarian cancer progression *in vivo.* **(A)** Female NSG mice inoculated i.p. with SKOV3- MUC16^ecto^-GFP-LUC tumor cells and subsequently treated with T-cells alone, or T-cells and MUC16^ecto^- BiTEDs were imaged for tumor burden over time. Tumor was injected on D0 i.p. T-cells were injected on D7 i.v in all animals. Animals that received BiTEDs treated were dosed i.p on D7, D9, D11, D14, D16 and D18. **(B)** Quantification of bioluminescence from **(A)**. **(C)** Female NSG tumor bearing mice were treated with T-cells alone or T-cells and MUC16^ecto^- BiTEDs. Serum cytokines were evaluated 7-days after treatment. Data shown are pooled results from 2 independent experiments with at least 3 independent donors. Data are plotted as mean ± SEM. *p < 0.01, **p < 0.05. ns, not significant.

To evaluate the *in vivo* efficacy of MUC16^ecto^-directed bispecific T-cell engagers, we intraperitoneally (i.p) injected female NSG mice with 3 x 10^6^ SKOV3 tumor cells modified to express MUC-16^ecto^ and luciferase. Seven days after tumor inoculation, mice were either untreated, treated with intravenous (i.v) activated human T-cells alone, or i.v T-cells and MUC16^ecto^-BiTEDs. Tumor-bearing mice treated with MUC16^ecto^- BiTEDs received additional BiTEDs injections on days 9, 11, 14, 16, and 18 for a total of 6 doses over two weeks. Animals were imaged on days 14, 21, 28, and 42 after tumor injection. As shown in **Figures 4A, B**, MUC16^ecto^-BiTEDs delayed radiographic progression of disease compared to no treatment or treatment with T-cells alone. Using the same experimental model, we evaluated serum cytokines in mice treated with BiTEDs. Tumor-bearing mice treated with T-cells and BiTEDs showed significantly elevated levels of systemic IL-2, IFN-γ, TNF-α, and IL-10, seven days after treatment (**Figure 4C**). We did not find any significant elevations of serum IL-6, GM-CSF, IL-13, or IL-17a.

### MUC16^ecto^-BiTEDs Increase Survival as Monotherapy and in Combination With Immune Checkpoint Blockade

**Figure 5:**
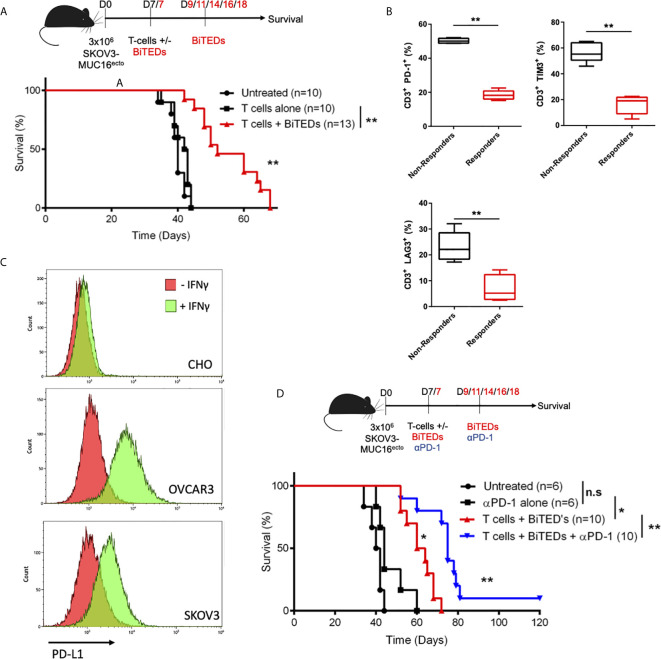
MUC16^ecto^-BiTEDs improve overall survival in tumor-bearing mice as monotherapy and in combination with PD-1 immune checkpoint blockade. **(A)** NSG tumor-bearing mice were inoculated i.p. with SKOV3-MUC16^ecto^ tumor cells and treated with either T-cells alone or T-cells and MUC16^ecto^-BiTEDs. Data shown are pooled results from 3 independent experiments with at least 4 independent donors. Data are plotted as mean ± SEM. **p < 0.05. Tumor was injected on D0 i.p. T-cells were injected on D7 i.v in all animals. Animals that received BiTEDs treated were dosed i.p on D7, D9, D11, D14, D16 and D18. **(B)** Immunophenotyping of human T-cells from the spleens of female NSG SKOV3-MUC16^ecto^ tumor-bearing mice treated with BiTEDs who had either succumbed to disease (Non-Responders) or from those who lived longer (Responders). **p < 0.05. **(C)** CHO, OVCAR3 and SKOV3 cells with or without IFN-γ stimulation for 24 hrs. Data shown representative of 2 independent experiments. **(D)** SKOV3-MUC16^ecto^ tumor-bearing mice were treated with T-cells and αPD-1 alone, T-cells and BiTEDs, or T-cells, αPD-1 and BiTEDs. Tumor was injected on D0 i.p. T-cells were injected on D7 i.v in all animals. Animals that received BiTEDs treated were dosed i.p on D7, D9, D11, D14, D16 and D18. Anti-PD-1 (clone EH12.2H7) was injected i.p. on D7, D14, D21 and D28. Data shown are pooled results from 3 independent experiments with at least 4 independent donors. Data are plotted as mean ± SEM. *p < 0.05 T-cells + αPD-1 vs T-cells + BiTEDs, **p < 0.05 T-cells + BiTEDs vs T-cells + BiTEDs + αPD-1. Statistical analysis for **(A, C)** performed using a log-rank (Mantel-Cox) test. ns, not significant.

Next, we asked if treatment with MUC16^ecto^-BiTEDs could prolong survival in tumor-bearing mice. Compared to treatment with T-cells alone, the administration of MUC16^ecto^-BiTEDs led to significantly increased survival in SKOV3-MUC16^ecto^ tumor-bearing mice (median OS; 42.5 days vs. 52 days, **p < 0.005) (**Figure 5A**). Since the tumor model we used for this experiment has sustained levels of MUC16 expression, immune escape by antigen loss or downregulation is an unlikely mechanism of disease progression. To better understand the potential mechanisms for BiTEDs treatment failure, we harvested spleens from BiTED-treated animals that succumbed early to disease and compared human T-cell phenotypes with spleens from responders. Responders were defined as treated animals living beyond 55 days. We found an increased proportion of CD3+ human T-cells expressing PD-1, TIM-3, and LAG3, markers associated with T-cell dysfunction, in non-responders compared to animals that responded better to therapy (**Figure 5B**). This suggests that some degree of T-cell dysfunction underlies BiTEDs treatment failure. It has been shown that ovarian cancer cells can upregulate PD-L1 in response to IFN-γ, a major inflammatory cytokine secreted by activated T-cells (33, 34). To test the hypothesis that PD-L1-mediated inhibition plays a significant role in attenuating immune responses in our tumor model, we first validated that both SKOV3 and OVCAR3 cell lines used in our experiments upregulate PD-L1 in the presence of IFN-γ (**Figure 5C**). CHO cells were used as negative controls. Next, we combined anti-PD-1 (αPD-1) immune checkpoint inhibition with MUC16^ecto^-BiTEDs and found that the combination significantly improved survival (median OS; 62 *vs.* 75 days, **p < 0.05), including the proportion of animals surviving beyond 100 days (**Figure 5D**). Treatment with MUC16^ecto^-BiTEDs was unsurprisingly superior to αPD-1 therapy alone (median OS; 62 *vs.* 44 days, **p < 0.05) (**Figure 5D**).

### Inhibition of Tumor-Angiogenesis Significantly Improves MUC16^ecto^-BiTEDs Immunotherapy

**Figure 6:**
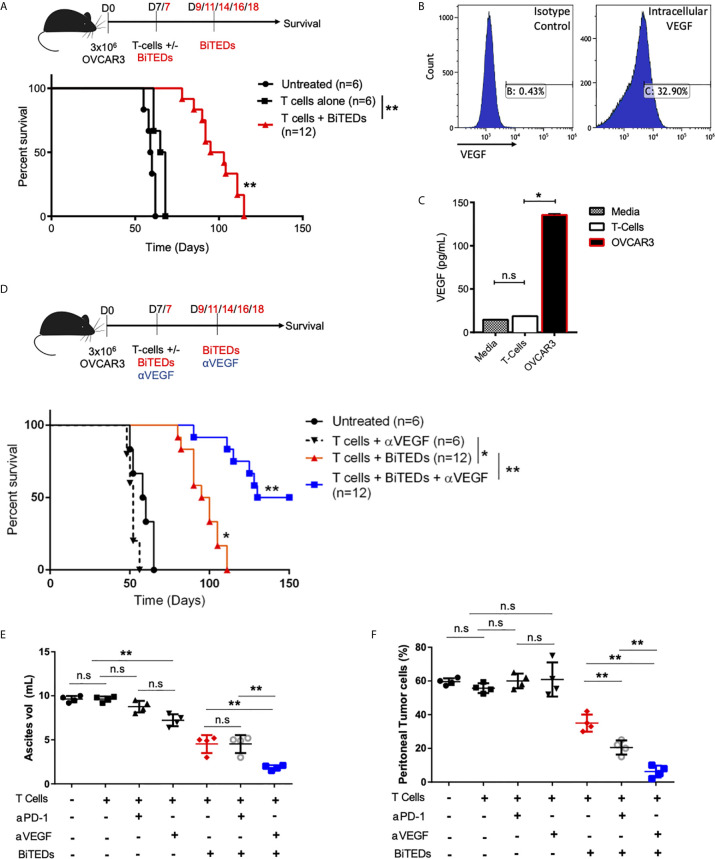
MUC16^ecto^-BiTEDs in combination with VEGF inhibition significantly improves ascites, peritoneal tumor burden, and overall survival. **(A)** Female NSG mice were inoculated i.p with OVCAR3 tumor cells and treated with T-cells alone or T-cells and MUC16^ecto^- BiTEDs. Data shown are pooled results from 2 independent experiments with at least 4 independent Leukopak donors. Data are plotted as mean ± SEM. **p < 0.05. Tumor was injected on D0 i.p. T-cells were injected on D7 i.v in all animals. Animals that received BiTEDs treated were dosed i.p on D7, D9, D11, D14, D16 and D18. **(B)** Expression level of VEGF in OVCAR3 cells by flow cytometry. **(C)** Evaluation of VEGF in supernatant collected from T-cells and OVCAR3. T-cells vs OVCAR3 (*p < 0.05). **(D)** OVCAR3 tumor-bearing mice treated with T-cells + αVEGF, T-cells + BiTEDs, or T-cells + BiTEDs + αVEGF. Data are plotted as mean ± SEM. T cells + αVEGF vs T cells + BiTEDs (*p < 0.005). T cells + BiTEDs vs T cells + BiTEDs + αVEGF (**p < 0.005). Statistical analysis for (a) and (d) performed using a log-rank (Mantel-Cox) test. Tumor was injected on D0 i.p. T-cells were injected on D7 i.v in all animals. Animals that received BiTEDs treated were dosed i.p on D7, D9, D11, D14, D16 and D18. Anti-VEGF (clone hvegf-mab1) was injected i.p. on D7, D11, D14, D18, and D21. **(E)** Volume of ascites collected from OVCAR3 tumor-bearing mice treated with T-cells alone, T-cells + αPD-1, T-cells + αVEGF, T-cells + BiTEDs, T-cells + αPD-1 + BiTEDs, or T-cells + αVEGF + BiTEDs. Data shown are pooled results from 2 independent experiments with at least 2 independent Leukopak donors. Data are plotted as mean ± SEM. **p < 0.05. **(F)** Percentage of MUC16^POS^, CD45^-^ peritoneal tumor cells corresponding to the same conditions as in **(E)**. Data are plotted as mean ± SEM. **p < 0.05. ns, not significant.

The critical role of angiogenesis in ovarian cancer has been extensively described (35). Increased VEGF expression is a poor prognostic indicator in ovarian cancer (36), and monoclonal antibodies against VEGF play an essential role in the clinical management of this disease (37). Further, VEGF inhibition has been shown to reduce ascites (38), a well-described immunosuppressive tumor microenvironment (39). OVCAR3 cells have been shown to secrete VEGF (40) and we hypothesized that combining MUC16^ecto^-BiTEDs with anti-VEGF (αVEGF) antibodies would substantially improve efficacy over MUC16^ecto^-BiTEDs monotherapy. First, we implanted female NSG mice i.p. with OVCAR3 cells and showed that treatment with MUC16^ecto^-BiTEDs improved survival over the infusion of T-cells alone (**Figure 6A**) (median OS; 66.5 vs. 99 days, **p < 0.005). We verified that the OVCAR3 cells used in these experiments expressed VEGF by intracellular flow cytometry (**Figure 6B**) and ELISA (**Figure 6C**). Next, we treated OVCAR3 tumor-bearing mice with MUC16^ecto^-BiTEDs and αVEGF therapy. In combination with VEGF inhibition, MUC16^ecto^-BiTEDs led to significant improvements in overall survival over BiTEDs treatment alone (median OS; 140 *vs.* 97.5 days, **p < 0.005) (**Figure 6D**). Treatment with T-cells plus αVEGF was inferior to therapy with MUC16^ecto^-BiTEDs (median OS; 97.5 *vs.* 59 days, *p < 0.005), suggesting that inhibition of angiogenesis alone is insufficient without concomitant cytotoxic T-cell engagement. Concordantly, we found significantly decreased ascites in mice treated with BiTEDs/αVEGF combination compared to BiTEDs/αPD-1 and BiTEDs monotherapy (**Figure 6E**). Evaluation of peritoneal tumor cells showed significantly decreased tumor burden with BiTEDs/αVEGF combination compared to BiTEDs/αPD-1 and BiTEDs monotherapy (**Figure 6F**). Although mice treated with αVEGF had decreased ascites (**Figure 6E**), there was no decrease in peritoneal tumor cells (**Figure 6F**), explaining the lack of survival benefit with this combination (**Figure 6D**).

## Discussion

In this study, we successfully generate and validate human taFv, scFv_2_ BiTEs specific for MUC16^ecto^ *in vitro* and *in vivo*. MUC16^ecto^-BiTEDs facilitate cytotoxicity in the presence of human T-cells and MUC16^pos^ ovarian cancer cells *in vitro*. Treatment of tumor-bearing mice with MUC16^ecto^- BiTEDs increases *in vivo* cytokine secretion, substantially delays tumor progression, and increases overall survival in xenograft preclinical murine models of ovarian peritoneal carcinomatosis.

In contrast to our previously reported CAR-based approach to MUC16^ecto^ positive ovarian cancer (24), MUC16^ecto^-BiTEDs have three distinct advantages. First, the scFv for MUC16^ecto^- BiTEDs were derived from human phage display libraries, limiting the potential for HAMA. Also, MUC16^ecto^-BiTEDs can easily be manufactured and administered without the intricate and labor-intensive manufacturing process required for CAR T-cells. Lastly, BiTEDs are relatively well-tolerated, and blinatumomab, a CD19- bispecific antibody, is currently FDA approved to treat B-cell hematologic malignancies (14). Because BiTEs redirect polyclonal T cells to the tumor, this creates the potential to generate TILs in patients who might otherwise have immunologically “cold” tumors. Furthermore, BiTEs have been reported to induce epitope spreading (15). The ability to promote neoantigen recognition significantly mitigates the potential for immune escape since solid tumors typically display surface antigen expression heterogeneity (41). These advantages could be exploited *via* combination therapy with other rationally-selected agents such as immune checkpoint inhibitors, anti-angiogenic agents, and immunogenic cytotoxic chemotherapy (42). A recent report by Crawford et al. (43) described the design and characterization of REGN4018, a MUC16-directed effector function-minimized bispecific antibody for the treatment of ovarian cancer. REGN4018 also binds the membrane-proximal portion of MUC16 (up to the fifth sea urchin sperm protein enterokinase and agrin domain) and retains efficacy in the presence of elevated levels of CA-125. REGN4018 was efficacious in both xenograft and syngeneic genetically modified mouse models, and combination with anti-PD-1 immune checkpoint blockade further enhanced efficacy. Notably, the authors showed that REGN4018 was well tolerated in mice and cynomolgus monkeys and it is undergoing clinical trial testing (NCT03564340). While both studies target different regions of the MUC16 extracellular domain, our MUC16^ecto^-BiTEDs are also efficacious *in vitro* and *in vivo*. Reassuringly, we also detected increases in IL-2, and IFN-γ, with attendant survival benefits in tumor-bearing mice. Both studies also support the addition of anti-PD-1 immune checkpoint blockade to further enhance bispecific T-cell engager efficacy. This report builds upon previously published literature in two important ways. We used multiple ovarian cancer cell lines for our initial screening, including isogenic MUC16^Pos^ SKOV3 and OVCAR3 cells. Testing the efficacy of MUC16^ecto^- BiTEDs against different cell lines minimizes the possibility that the cytotoxic effects observed are cell line restricted. While both studies outline a role for combination with anti-PD-1, this study goes further by demonstrating the potency of concurrent VEGF inhibition with MUC16^ecto^-BiTEDs therapy. The MUC16-directed tandem linked single chain variable fragment bispecific detailed in this manuscript has a KD of 70 nM, and although it shows single-agent efficacy *in vitro* and *in vivo*, there is room for further optimization. In addition, this BiTE configuration, lacking a stabilized Fc domain is prone to relatively short half-life *via* renal clearance (8). To address the former, ongoing work is aimed at solving the crystal structure of MUC16^ecto^ bound to our scFv. Using this structural basis, informed amino acid substitutions can be made to increase affinity. Another approach we are currently evaluating is combining our current MUC16^ecto^-BiTEDs with MUC16^ecto^-specific armored CAR T-cells. These armored CAR T-cells will target MUC16^ecto^ and secrete MUC16^ecto^-BiTEDs in the tumor microenvironment. Not only would this lead to sustained levels of MUC16^ecto^-BiTEDs in the TME, but these BiTEs would also recruit endogenous T-cells and potentially promote antigen spreading. This approach has been shown to be efficacious in Glioblastoma preclinical mouse models using an EGFRvIII-targeting CAR T-cell modified to secrete EGFR BiTEs in the tumor microenvironment (44).

VEGF inhibition plays an important clinical role in managing newly diagnosed, platinum-sensitive, and platinum-resistant ovarian cancer (45). VEGF-A has been shown to directly upregulate PD-1 expression on T-cells and promote an “exhausted” immunophenotype (46). It was demonstrated that VEGF inhibition induced PD-1 expression on T-cells, and dual blockade of PD-1 and VEGF led to significant tumor regression in a preclinical colon cancer model. This could potentially explain our finding of increased PD-1, TIM3, and LAG3 in T-cells recovered from BiTEDs-treated animals that succumbed to disease. Yet blockade of the PD-1 immune checkpoint only partly rescues this phenotype, suggesting the role of multiple inhibitory pathways. Inhibition of VEGF signaling in the tumor microenvironment has been reported to improve cytotoxic CD8+ T-cell activity *via* upregulation of cytokine secretion both as monotherapy and in combination with immune checkpoint blockade (47). As BiTE directly rely on T-cells to exert their antitumor activity, it was unsurprising that PD-1 upregulation was one of the mechanisms leading to treatment failure and that subsequent blockade of PD-1/L1 reversed this phenotype. The magnitude to which VEGF blockade improved MUC16^ecto^-BITED efficacy was surprising. We found that peritoneal tumor burden was significantly less in tumor-bearing mice treated with MUC16^ecto^-BiTEDs and αVEGF compared to mice treated with MUC16^ecto^-BiTEDs/αPD-1. One potential explanation for improved survival in animals treated with αVEGF over αPD-1 could be because VEGF inhibition decreases ascites and reduces T-cell dysfunction, leading to increased tumor debulking by T-cells. As the peritoneal tumor burden decreases, the amount of ascites further decreases, leading to further improvement in T-cell function.

MUC16^ecto^ is an ideal target for BiTE, antibody, or CAR-based immunotherapy due to its retention on the cell surface, ubiquity, and very low expression in non-Mullerian tissue. In a previous study we evaluated normal tissue expression of antibodies directed MUC16^ecto^ (23). MUC16^ecto^ expression was restricted to endocervix, luminal esophageal, crypts of the gastric glands, and pancreatic islets, and targeting the retained portion of MUC16 has been shown to be safe in preclinical syngeneic CAR T-cell mouse models (24). Crawford et al. (43) did not find any significant toxicity in genetically engineered murine and cynomolgus animal models treated with MUC16 BiTE. Phase 1 clinical trials are ongoing for both CAR T-cells (NCT02498912) and BiTE applications (NCT03564340). Besides combination therapy with αVEGF or αPD-1 antibodies, optimizations could also be made to the BiTEDs architecture to further improve stability or minimize the number of doses required for therapeutic efficacy (48). To our knowledge, this is the first report of a human bispecific tandem linked single chain variable fragment targeting the retained portion of MUC16 (MUC16^ecto^). This preclinical report demonstrates the feasibility and efficacy of MUC16^ecto^-BiTEDs and provides rationale for combination with αVEGF therapy for ovarian cancer.

## Data Availability Statement

The original contributions presented in the study are included in the article/supplementary material. Further inquiries can be directed to the corresponding author.

## Ethics Statement

All murine studies were done in accordance with Memorial Sloan Kettering Cancer Center Institutional Animal Care and Use Committee approved protocol (00–05–065).

## Author Contributions

OOY, PW, RJB and DS designed the experiments, interpreted the results and wrote the manuscript. TDR, IL, AK, PW, ZC, HL, and TJP designed, performed and/or analyzed the experiments.

## Funding

National Institutes of Health, National Cancer Institute grant P01 CA190174 (DS and OOY). Executive Committee on Research Physician Scientist Development Award (OOY). US National Institutes of Health grants 5 P01 CA190174-03 and 5 P50 CA192937-02 (RJB).

## Conflict of Interest

Authors PW, ZC, and HL were employed by Eureka Therapeutics Inc. DS, OOY and Memorial Sloan Kettering Cancer Center have submitted a patent application for this antibody and the related bispecific construct. RJB is a co-founder of and receives royalties from Juno Therapeutics.

The remaining authors declare that the research was conducted in the absence of any commercial or financial relationships that could be construed as a potential conflict of interest.

## Abbreviations

BiTEs, Bispecific T-cell Engagers; taFv, scFv_2_, Bispecific tandem linked single chain variable fragments; BiTEDs, MUC16^ecto^ bispecific tandem single-chain variable fragments; EOC, Epithelial Ovarian Cancer; TILs, Tumor-infiltrating T-cells; scFv, Single chain variable fragments; TCR, T-cell innate antigen-specific receptor; CAR, Chimeric Antigen Receptor; MUC16, Mucin-16; LDH, Lactate Dehydrogenase; HAMA, Human anti-mouse antibody; i.v, Intravenous; i.p, Intraperitoneal; IL-2, Interleukin-2; IFN-γ, interferon-gamma; PD-1, programmed cell death- 1; VEGF, Vascular endothelial growth factor.
